# Women in Cardiovascular Surgery: Diversity as a Strategy for the Future

**DOI:** 10.21470/1678-9741-2026-0066

**Published:** 2026-09-08

**Authors:** Magaly Arrais dos Santos, Amanda Arrais Bento de Souza

**Affiliations:** 1 Cardiovascular Surgery, Hospital do Coração (Hcor), São Paulo, São Paulo, Brazil.; 2 Department of Cardiovascular Surgery, Santa Casa de Misericórdia de Porto Alegre, Porto Alegre, Rio Grande do Sul, Brazil.

**Figure:**
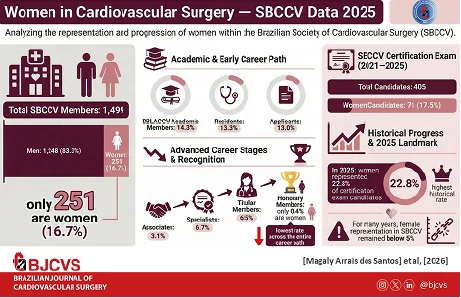

Cardiovascular surgery has historically been established as a specialty of high technical complexity, characterized by long training periods, strong hierarchical structures, and intense clinical responsibility. Despite the progressive feminization of medicine overall, cardiovascular surgery remains among the specialties with the lowest female representation, both in Brazil and internationally^[1,2]^. This disparity should not be viewed merely as a historical legacy, but rather as a warning sign regarding the future and sustainability of the specialty itself.

The trajectory of women in cardiovascular surgery has been marked by significant achievements attained in adverse contexts. In 1960, Dr. Nina Starr Braunwald broke paradigms by performing the frst open-heart surgery conducted by a woman and by making decisive contributions to the development of prosthetic heart valves^[3]^. In Brazil, pioneers such as Dr. Dirce da Costa Zerbini played a central role in the consolidation of cardiopulmonary bypass and the technical advancement of national cardiac surgery^[4]^. In the following decades, surgeons like Dr. Vera Lúcia Amaral Molari Piccardi integrated the frst groups of women formally trained in the specialty, opening institutional paths that were previously non-existent.

Despite this legacy, records from the Brazilian Society of Cardiovascular Surgery (SBCCV) show that for many years female representation among its members remained below 5%. Recent data indicate that, in 2025, the SBCCV has 1,499 members, of whom only 251 are women, corresponding to approximately 16.7% of the total (Central Figure)^[5]^. The membership is divided into the following categories with their respective percentages of women: academic members (DBLACCV), 14.3%; residents, 13.3%; applicants, 13.0%; associates, 3.1%; specialists, 6.7%; titular members, 6.5%; retired members, 9.8%; and honorary members, 0.4%. Although this represents progress compared with previous decades, the distribution highlights a concentration of women in the early stages of training and professional development, with a marked decline at higher career levels, reflecting a persistent pattern of structural underrepresentation.

This imbalance becomes even more evident when analyzing the so-called leadership bottleneck. Although women already constitute the majority of medical students, a progressive “funnel” limits their entry, retention, and advancement in cardiovascular surgery^[6]^. Underrepresentation is particularly pronounced in leadership roles, service coordination positions, senior academic appointments, and first authorship of scientific publications^[7]^. This phenomenon reflects less a lack of interest or aptitude and more the persistence of structural barriers, including wage inequality, implicit bias in performance evaluation, and limited access to mentorship and professional sponsorship networks^[8]^. National data further corroborate this pattern. The Medical Demographics in Brazil 2025 report, published by the Brazilian Ministry of Health and coordinated by Scheffer, demonstrates that the distribution of medical specialists by sex and age reveals a progressive decline in female representation across surgical specialties as age and seniority increase, with cardiovascular surgery remaining markedly male-dominated^[9]^.

In the Brazilian context, this reality is exemplified by the fact that, despite the large number of cardiovascular surgery training centers nationwide, only a very limited number are currently led by women, notably Dr. Marcela Salles, Head of the Cardiovascular Surgery Service at Santa Casa de Porto Alegre, and Dr. Valquíria Pelliser Campagnucci, Head of the Cardiovascular Surgery Service at Santa Casa de São Paulo, highlighting the exceptional, rather than normative, nature of female leadership within the specialty. Within this context, the specialist certification examination of the SBCCV emerges as a relevant marker not only of technical competence but also of career progression. Between 2021 and 2025, of the 405 candidates registered for the examination, only 71 were women, representing approximately 17.5% of the total^[5]^. Until 2024, female participation remained proportionally low in both applications and approvals. In 2025, however, a significant increase was observed, with women accounting for approximately 22.8% of applicants and a higher absolute number of approvals, suggesting that access to full certification may respond positively when structural barriers are reduced. Nevertheless, certification remains a critical bottleneck in professional advancement and in the persistent underrepresentation of women at the highest levels of the specialty.

Contemporary scientific evidence has consistently demonstrated that diversity does not compromise excellence. Large population-based studies indicate that female surgeons achieve clinical outcomes equivalent to, and in certain scenarios superior to, those of their male counterparts, including lower rates of mortality and postoperative complications^[10,11]^. These findings shift the debate from symbolic representation to the domain of quality of care, reinforcing that female inclusion is not a social concession, but rather a factor associated with safer practices, more effective communication, and greater adherence to clinical protocols.

In light of this scenario, it becomes clear that the evolution of cardiovascular surgery cannot rely on spontaneous or exclusively individual changes. Institutional intentionality is required. Structured mentorship programs, transparent career progression policies, pay equity, and safe and inclusive work environments are essential tools to attract, retain, and develop talent^[12]^. Scientific societies such as the SBCCV play a central role in this process, both through their organizational capacity and their symbolic power to legitimize transformation.

Valuing and expanding the presence of women in cardiovascular surgery represents a commitment to the sustainability of the specialty. Acknowledging the past, confronting present challenges, and investing in equity are not ideological choices, but necessary strategies to ensure that cardiovascular surgery remains at the forefront of medicine, combining technical excellence, institutional maturity, and responsibility toward the future.
